# The effect of uterine entry technique on chorioamniotic membrane separation in fetoscopic laser photocoagulation for twin-to-twin transfusion syndrome: protocol for a randomized controlled trial

**DOI:** 10.1186/s12884-025-08410-5

**Published:** 2025-11-21

**Authors:** Brian A. Burnett, Jessian L. Munoz, Rebecca M. Johnson, Christian M. Parobek, Luis E. Delgadillo Chabolla, Cara Buskmiller, Roopali V. Donepudi, Magdalena Sanz Cortes, Michael A. Belfort, Ahmed A. Nassr

**Affiliations:** Department of Obstetrics and Gynecology, Baylor College of Medicine and Texas Children’s Fetal Center, 6651 S Main St, Houston, TX 77030 USA

**Keywords:** Chorioamniotic separation, Fetoscopic laser photocoagulation, Twin pregnancy, Twin-to-twin transfusion syndrome

## Abstract

**Background:**

Chorioamniotic membrane separation (CAS) is a recognized complication of fetoscopic laser photocoagulation (FLP) for twin-to-twin transfusion syndrome (TTTS), associated with increased risks of preterm prelabor rupture of membranes (PPROM) and preterm birth (PTB). Although CAS is well described, its incidence and relationship to specific surgical techniques, particularly the method of uterine entry, are not well defined in the published literature. No randomized trials have evaluated whether entry technique influences the risk of CAS.

**Methods:**

We present the protocol for a randomized controlled trial comparing sharp-trocar and Seldinger uterine entry techniques for FLP in TTTS diagnosed between 16 + 0 and 26 + 0 weeks of gestation. The primary outcome is CAS diagnosed intraoperatively or within 48 h postoperatively by ultrasound evaluation. Secondary outcomes include preterm birth, procedure-to-delivery latency, perinatal survival, and maternal complications. A total of 216 participants will be enrolled, analyses will be conducted on an intention-to-treat basis.

**Discussion:**

This trial will provide the first randomized evidence on whether uterine entry technique affects the incidence of CAS and perinatal outcomes in TTTS, with the potential to guide surgical best practices in fetal therapy.

**Trial registration:**

February 17, 2025 at ClinicalTrials.gov, NCT06829901.

## Background

Twin-to-twin transfusion syndrome (TTTS) complicates approximately 10–15% of monochorionic diamniotic twin pregnancies [1]. It results from unbalanced blood flow across shared placental vascular anastomoses, producing hypovolemia and oliguria in the “donor” twin, and volume overload with polyuria in the “recipient” [2, 3]. The syndrome is associated with progressive fetal cardiovascular compromise and substantial perinatal morbidity and mortality [4–6]. 

Diagnosis of TTTS is established by ultrasound when there is oligohydramnios in the donor sac (deepest vertical pocket [DVP] < 2 cm) together with polyhydramnios in the recipient sac (DVP >8 cm) [1, 5, 7]. Disease severity is classified using the Quintero staging system, which stratifies TTTS based on progressive sonographic and physiologic findings [7]. Stage I is defined by fluid discordance with visible bladders in both twins; Stage II by non-visualization of the donor bladder; Stage III by critically abnormal Doppler studies; Stage IV by hydrops fetalis; and Stage V by intrauterine demise of one or both twins. This framework provides a standardized approach for prognosis and management.

For Stage II–IV TTTS presenting between 16 and 26 weeks of gestation, fetoscopic laser photocoagulation (FLP) of placental vascular connections is the treatment of choice. Compared with serial amnioreduction or expectant management, this approach provides superior survival outcomes and improved long-term neurodevelopment [4, 5, 8–10]. Although Stage I TTTS is typically managed expectantly, FLP may be considered after individualized assessment, particularly for severe maternal symptoms attributable to polyhydramnios or progressive cervical shortening [5, 11]. 

Despite its benefits, FLP carries procedure-related risks, including preterm prelabor rupture of membranes (PPROM), preterm birth (PTB), septostomy, and chorioamniotic membrane separation (CAS). Severe maternal complications are rare, occurring in fewer than 2% of fetoscopic procedures [12]. Among these complications, iatrogenic CAS, defined as detachment of the amnion from chorion as a direct result of fetal intervention, is reported in 7.5–20.7% of pregnancies following FLP and is associated with increased risks of PPROM, earlier delivery, and neonatal morbidity [13–17]. 

In a systematic review and meta-analysis of 1,881 cases of FLP, CAS was associated with significantly higher odds of PPROM before 34 weeks (OR 3.98, 95% CI 1.76–9.03; *p* < 0.001), PTB before 32 weeks (OR 1.80, 95% CI 1.16–2.80; *p* = 0.008), and reduced neonatal survival (OR 0.41, 95% CI 0.24–0.70; *p* = 0.001). CAS was also associated with earlier gestational age at intervention (mean difference − 1.07 weeks, 95% CI − 1.89 to − 0.24; *p* = 0.01) and delivery (mean difference − 1.74 weeks, 95% CI − 3.13 to − 0.34; *p* = 0.01) [13–19]. 

The pathogenesis of CAS is likely multifactorial. Most cases originate at the cannula insertion site, where disruption of the fused membranes permits amniotic fluid to dissect between layers [17]. At earlier gestational ages, incomplete chorion-amnion fusion leaves the membranes more vulnerable to disruption [20]. Injury is unlikely to heal because the amnion lacks vasculature, resulting in a tendency for persistent separation [21, 22]. 

Access to the amniotic cavity for FLP can be achieved using either a multistep Seldinger approach, involving needle puncture with guidewire passage and cannula advancement, or alternatively, the cannula may be introduced directly over a sharp trocar in a single step. The two approaches may differ in the extent of tenting and tangential stress applied to the membranes [23]. The relationship between entry technique and CAS risk has not been well characterized, and no randomized trial has directly compared these approaches.

## Methods

### Study design

This is a prospective, single-blind randomized controlled trial (RCT) comparing direct trocar entry against Seldinger technique for fetoscopic laser photocoagulation in twin-to-twin transfusion syndrome. This study protocol has been developed in accordance with the Standard Protocol Items: Recommendations for Interventional Trials (SPIRIT) checklist. No patient or public involvement occurred in the design, conduct, reporting, or dissemination of this trial.

### Study setting

The trial is being sponsored by Texas Children’s Hospital, with clinical sites in Houston and Austin, Texas, USA. The study has been approved by Baylor College of Medicine IRB (H-56175).

### Eligibility criteria

#### Inclusion criteria

Pregnant patients aged ≥ 18 years with monochorionic–diamniotic twin pregnancies complicated by Quintero stage I–IV twin-to-twin transfusion syndrome, scheduled to undergo fetoscopic selective laser photocoagulation at Texas Children’s Hospital between 16 + 0 and 26 + 0 weeks’ gestation, and able to provide informed consent are eligible for enrollment.

### Exclusion criteria

Patients are excluded if they decline FLP, have triplet or higher-order multiple gestations, are outside the gestational age range at the time of surgery, have a cervical length < 1.5 cm, or have a known subchorionic hematoma.

### Intervention

#### Surgical technique

Fetoscopic laser photocoagulation (FLP) will be performed by experienced fetal surgeons using a standardized approach under maternal local anesthesia (lidocaine) and intravenous sedation (midazolam and fentanyl). Both entry techniques will be performed under continuous ultrasound guidance.

In the Seldinger-entry group, an 18-gauge needle will be introduced into the recipient’s amniotic sac. A guidewire will be advanced through the needle, and a Teflon cannula (Cook^®^ Medical Inc., Bloomington, IN, USA) over a blunt trocar will be inserted along the guidewire tract through the abdominal and uterine walls into the recipient sac.

In the sharp trocar group, the cannula (Cook^®^ Medical Inc., Bloomington, IN, USA) with an integrated sharp trocar will be inserted directly through the abdominal and uterine walls into the recipient sac without prior guidewire placement.

After entry, a fetoscope will be introduced through a 9, 10, or 12 French cannula based on the surgeon’s clinical discretion. The placental vascular equator will be mapped systematically, and selective, non-sequential photocoagulation of communicating placental vessels will be performed using a diode laser. To complete the laser photocoagulation, the “Solomon technique” is applied, creating a continuous line of laser ablation along the vascular equator to ensure complete dichorionization [24]. At the conclusion of the procedure, an amnioreduction from the recipient’s sac is performed to restore physiologic amniotic fluid volume. The quantity removed varies depending on the degree of preoperative polyhydramnios, with the goal of achieving a normal deepest vertical pocket in the recipient sac. When clinically indicated and at surgeon discretion, an absorbable gelatin plug is placed in the uterus to seal the cannula tract and reduce fluid leakage and membrane disruption. Intraoperative complications may necessitate modification or discontinuation of the entry technique; such deviations will be documented prospectively.

### Outcomes

The primary outcome is the incidence of immediate chorioamniotic membrane separation (CAS), diagnosed by standardized postoperative ultrasound performed 24 to 48 h after fetoscopic laser photocoagulation. The acquisition protocol includes targeted sweeps of the membrane interface, imaging of the cannula insertion site, deepest vertical pocket measurements in each sac, placental cord insertions, and assessment of overall membrane contour. In this trial, CAS is categorized by extent of detachment of the amnion from the chorion as < 50% or ≥ 50% of the intrauterine cavity.

Secondary outcomes include delayed CAS (first detected more than 48 h postoperatively), gestational age at delivery, preterm birth (< 34 and < 32 weeks), procedure-to-delivery interval in weeks, placental abruption, intra-amniotic infection, PPROM, maternal hospital admission secondary to CAS, and neonatal survival to hospital (NICU) discharge. Ultrasound follow-up will occur weekly until 6 weeks postoperatively, and every two weeks thereafter unless more frequent follow up is clinically indicated. All adverse events and serious adverse events, including maternal-fetal complications will be recorded and reported to the IRB in line with institutional policy.

### Sample size

**Fig. 1:**
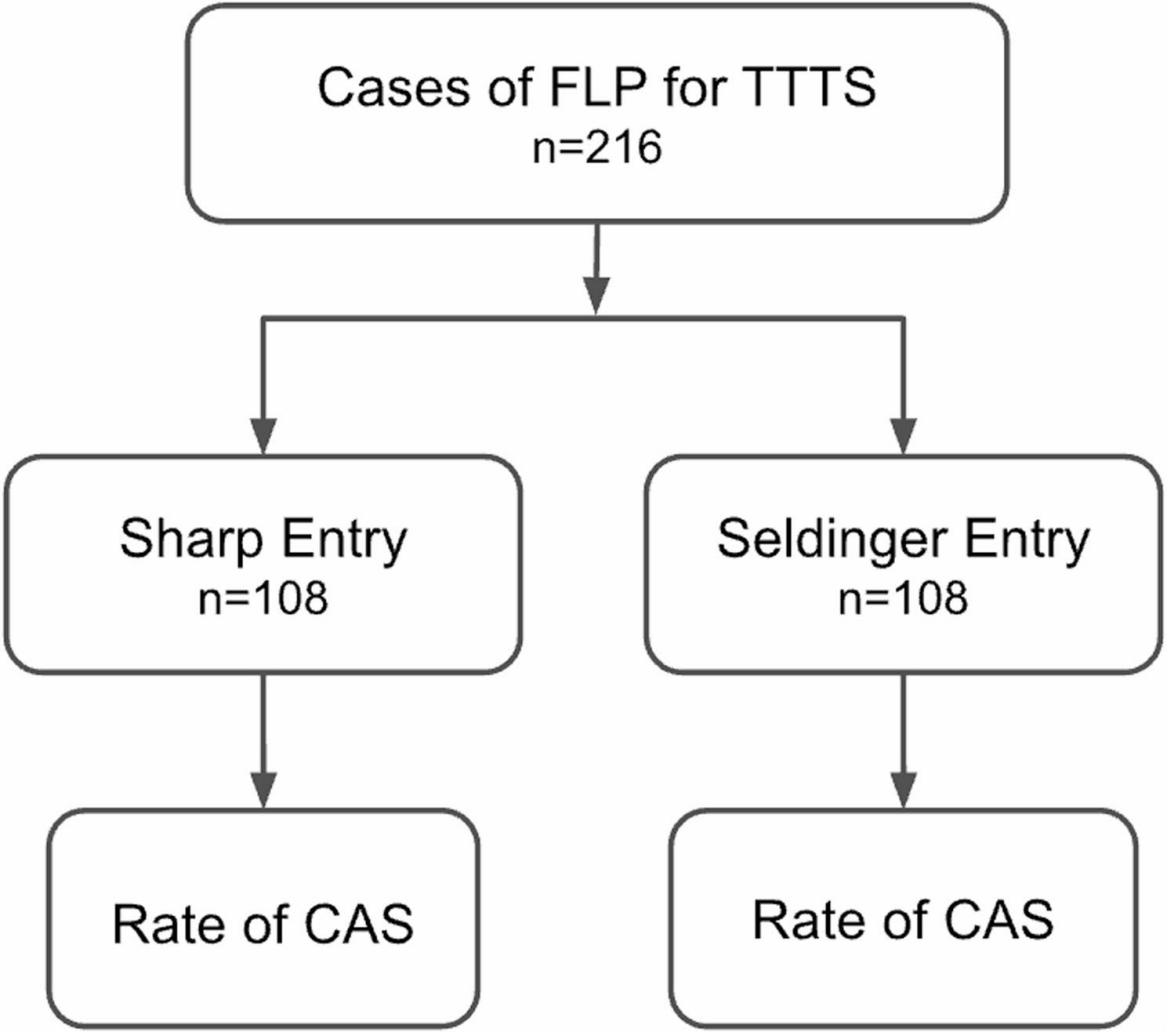
Study flow diagram. Random allocation of cases of fetoscopic laser photocoagulation (FLP) for twin-to-twin transfusion syndrome (TTTS) to sharp trocar versus Seldinger entry groups, with subsequent assessment of chorioamniotic membrane separation (CAS)

The required sample size is 216 participants (108 per arm), calculated for two independent study groups with dichotomous outcomes to detect a clinically meaningful difference between entry techniques (Fig. 1). Assumptions included an anticipated incidence of CAS of 7.5% in group 1 and 20.7% in group 2, with a two-sided α of 0.05 and β of 0.20 (80% power). These incidence estimates were derived from a recent systematic review and meta-analysis, in which the lowest reported rate of CAS was 7.5% (Bergh et al.) and the highest was 20.7% (Ortiz et al.) [13, 15]. 

### Randomization and allocation concealment

Simple randomization without blocking will be performed in a 1:1 ratio using the National Institutes of Health (NIH) Randomization Tool. The generated allocation sequence is securely imported by an independent statistician into a password-protected REDCap randomization module, which executes assignments at the time of enrollment, concealing group allocation until after participant registration [25]. Access to the randomization sequence is limited to designated study personnel who are not involved in the recruitment, consenting, or randomization process (research manager and data analyst) to maintain rigorous allocation concealment and minimize the risk of selection bias.

### Blinding

Given the nature of the intervention, blinding of surgeons and procedural staff is not feasible; therefore, the trial will follow an open-label design for procedural conduct. Group assignment will be revealed only to the operating room team at the time of the procedure. The postoperative ultrasound at 24 to 48 h is reviewed by the operating surgeon for clinical care and is therefore unblinded. For study endpoint determination, all postoperative and follow-up images and cine clips, whether obtained at the sponsor site or externally, will be uploaded to a secure portal and assessed by a maternal-fetal medicine specialist blinded to group assignment, using standardized sonographic criteria. The blinded central assessment will be used for the primary endpoint (CAS) and for diagnosis and classification of delayed CAS based on the last ultrasound prior to delivery. Unblinding is unnecessary for clinical care, since treating physicians know the entry technique at surgery. All imaging datasets for central review will be coded with anonymized study identifiers to prevent inadvertent unblinding. Data analysts will remain blinded to group allocation throughout the statistical analysis.

### Data collection and management

All study data are being collected prospectively and entered into a secure, web-based Research Electronic Data Capture (REDCap) database hosted at Baylor College of Medicine. REDCap provides an encrypted, access-controlled environment that supports audit trails for tracking data entry and modifications [25]. Each participant will be assigned a unique study identifier, and identifiable information will be stored separately from clinical data to ensure de-identification. Only authorized study personnel will have access to the database, and regular data quality checks will be performed to ensure accuracy and completeness.

### Statistical methods

All analyses will follow the intention-to-treat principle, with participants analyzed in the groups to which they were randomized, regardless of protocol deviations or loss to follow-up. Categorical variables will be compared using the Chi-square test or Fisher’s exact test, as appropriate. Continuous variables will be assessed for normality and analyzed with the Student’s t-test for normally distributed data or the Mann–Whitney U test for non-normally distributed data. For dichotomous outcomes, adjusted risk ratios will be estimated using multivariable regression models with a log link and binomial distribution; if convergence issues arise, a Poisson model with robust variance will be applied.

Missing data will be minimized through rigorous follow-up procedures and data verification. If missing outcome data occur, a complete-case analysis will be the primary approach, supplemented by sensitivity analyses using multiple imputation under the assumption of missing at random. The extent and pattern of missing data will be reported.

Pre-specified subgroup analyses will assess whether the effect of uterine entry technique on CAS rates is consistent across clinically relevant strata. These will include gestational age at surgery (< 17 weeks vs. ≥ 17 weeks), as earlier gestational age is hypothesized to increase CAS risk due to thinner, less fused membranes, and placental location (anterior vs. posterior), as anterior placentation may increase CAS risk due to greater technical difficulty and membrane manipulation [26]. Interaction terms will be included in the statistical models to formally test for heterogeneity of treatment effect. A two-sided *p*-value < 0.05 will be considered statistically significant for both primary and subgroup analyses. No interim analyses or formal stopping rules are planned.

### Data and safety monitoring

The principal investigator, with co-investigators and research staff, reviews study data and adverse events quarterly to ensure safety and data integrity. Medical history, procedural details, and adverse events are documented, and summary reports are submitted to the Baylor College of Medicine IRB at continuing review.

## Discussion

This randomized controlled trial will address an important evidence gap regarding the impact of uterine entry technique on the development of CAS during FLP for TTTS. Although FLP is the established standard of care for mid-trimester Stage II-IV TTTS and is considered for select Stage I cases, its use is not without complications. CAS is a relatively common iatrogenic finding linked to PPROM, PTB, and lower neonatal survival, and minimizing its occurrence is an important objective in procedural optimization.

Current literature on CAS largely derives from retrospective series and single-center experiences, which are inherently limited by selection bias, heterogeneity in surgical approach, and variability in how CAS is diagnosed and reported. Entry-related factors, such as membrane manipulation and tenting during cannula insertion, may influence CAS risk between techniques. However, in the absence of randomized data, the impact of modifying uterine entry technique remains uncertain.

By prospectively comparing sharp trocar and Seldinger entry methods in a standardized surgical and postoperative care framework, this trial will provide high-quality evidence to inform best practices for uterine entry during FLP. If differences in CAS risk are demonstrated without introducing new risks, the findings may inform surgical practice and provide insights into strategies for optimizing gestational latency, PPROM prevention, and neonatal outcomes.

The relevance of this research extends beyond TTTS. Additional fetoscopic procedures, such as those performed for twin anemia–polycythemia sequence (TAPS), fetoscopic endoluminal tracheal occlusion (FETO), or amniotic band release, require mid-trimester uterine access, often under similar anatomic constraints. Findings from this study may therefore have broader applicability to procedural planning and training in fetal surgery.

Strengths of this trial include its randomized design, the use of clearly defined CAS diagnostic criteria, and blinded outcome assessment when feasible. Another strength is that procedures will be performed by a limited number of experienced fetal surgeons using a standardized technique, with multiple surgeons available to assist when needed. A limitation of the trial is the inability to blind operating surgeons to the assigned entry technique.

Ultimately, the results of this study have the potential to inform surgical practice in TTTS management by identifying whether entry technique impacts CAS, preterm birth, and survival outcomes.

### Trial status

Protocol version 1.0, dated 22 October 2024. Ethics approval was granted by the Baylor College of Medicine Institutional Review Board (IRB #H-56175) on 3 December 2024. Recruitment commenced on 14 March 2025 and is ongoing. The anticipated completion date is March 2028. This trial was registered on February 17, 2025 at ClinicalTrials.gov (NCT06829901).

## Data Availability

The datasets generated and/or analyzed during the current study are not publicly available due to participant privacy restrictions but are available from the corresponding author, Dr. Ahmed A. Nassr, on reasonable request. At the end of the trial, the original locked database will be securely retained at Texas Children’s Hospital.

## Abbreviations

CAS: Chorioamniotic membrane separation
DVP: Deepest vertical pocket
FETO: Fetoscopic endoluminal tracheal occlusion
FLP: Fetoscopic laser photocoagulation
IRB: Institutional Review Board
MCDA: Monochorionic diamniotic
OR: Odds ratio
PPROM: Preterm prelabor rupture of membranes
PTB: Preterm birth
RCT: Randomized controlled trial
TAPS: Twin anemia–polycythemia sequence
TTTS: Twin–to–twin transfusion syndrome
